# Single-Photon Avalanche Diode with Enhanced NIR-Sensitivity for Automotive LIDAR Systems

**DOI:** 10.3390/s16040459

**Published:** 2016-03-30

**Authors:** Isamu Takai, Hiroyuki Matsubara, Mineki Soga, Mitsuhiko Ohta, Masaru Ogawa, Tatsuya Yamashita

**Affiliations:** Toyota Central R&D Labs., Inc., 41-1, Yokomichi, Nagakute, Aichi 480-1192, Japan; hmatsu@mosk.tytlabs.co.jp (H.M.); msoga@mosk.tytlabs.co.jp (M.S.); ohtam@mosk.tytlabs.co.jp (M.O.); ogawa@mosk.tytlabs.co.jp (M.O.); tatsu-y@mosk.tytlabs.co.jp (T.Y.)

**Keywords:** avalanche photodiodes, light detection and ranging (LIDAR), single-photon avalanche diode (SPAD), time-of-flight (TOF), depth sensor, rangefinder, 3-D imaging, single-photon detector, advanced driver assistance system (ADAS)

## Abstract

A single-photon avalanche diode (SPAD) with enhanced near-infrared (NIR) sensitivity has been developed, based on 0.18 μm CMOS technology, for use in future automotive light detection and ranging (LIDAR) systems. The newly proposed SPAD operating in Geiger mode achieves a high NIR photon detection efficiency (PDE) without compromising the fill factor (FF) and a low breakdown voltage of approximately 20.5 V. These properties are obtained by employing two custom layers that are designed to provide a full-depletion layer with a high electric field profile. Experimental evaluation of the proposed SPAD reveals an FF of 33.1% and a PDE of 19.4% at 870 nm, which is the laser wavelength of our LIDAR system. The dark count rate (DCR) measurements shows that DCR levels of the proposed SPAD have a small effect on the ranging performance, even if the worst DCR (12.7 kcps) SPAD among the test samples is used. Furthermore, with an eye toward vehicle installations, the DCR is measured over a wide temperature range of 25–132 °C. The ranging experiment demonstrates that target distances are successfully measured in the distance range of 50–180 cm.

## 1. Introduction

Recently, advanced driver assistance systems (ADASs) have been designed and developed not only to make automobiles more comfortable, but also to reduce the number of traffic accidents [1]. Forward collision warning (FCW), autonomous emergency breaking (AEB), and pedestrian detection, to cite a few examples, rely on various technologically advanced sensors. High-accuracy ranging sensors are particularly important components of ADASs. Among ranging sensor technologies, millimeter-wave RADARs and stereo-vision cameras remain the key sensors of choice. However, these sensors do have limitations, so a number of ADAS implementations rely on the data fusion of two or more sensors.

In this context, we have been developing an optical long-range sensor technology based on the time-of-flight (TOF) principle for next-generation ADASs [2,3,4]. This light detection and ranging (LIDAR) sensor offers a very good balance of overall performance in terms of spatial (image) resolution, field-of-view, precision, and depth range. In our LIDAR sensor, avalanche photodiodes operating in so-called Geiger mode have been employed as photodetectors (*i.e.*, optical receivers). The device, referred to as a single-photon avalanche diode (SPAD), is a highly sensitive photodetector capable of outputting a precise and digital trigger signal upon the detection of ultralow-power signals, down to the single photon level. Moreover, since the SPAD can be fabricated using general complementary metal-oxide-semiconductor (CMOS) technology, SPADs, their front-end circuits, and a complete digital signal processor (DSP) have been successfully and integrally designed as a low-cost system-on-a-chip implementation. Our latest LIDAR system [3] using the CMOS SPAD technology has achieved 100-m ranging even under the strong outdoor lighting environment of 70 klux.

Although the SPAD is sensitive to a single photon, not all photons are detected. For example, photons that penetrate the SPAD are not detected. Also, photo-generated electrons do not always trigger an avalanche breakdown because it is a probabilistic process. Thus, a higher photon detection efficiency (PDE) is crucial for any sensing application. Until recently, CMOS SPAD technologies have shown a high PDE only in the visible light band. However, in the near-infrared (NIR) band between 800 nm and 1 μm, CMOS SPAD devices have exhibited rather low PDEs. For example, at the wavelength of interest in our application (870 nm), the PDEs have typically been below 5%. In order to increase the measurement range and/or rate of automotive LIDAR systems that generally employ NIR light sources, a much higher PDE in the NIR band is highly desirable. More generally, however, the ranging performance depends on the overall sensitivity, which, in turn, is characterized by the product of the PDE and the fill factor (FF) of the SPAD. Therefore, key to realizing a high-performance automotive LIDAR system is having both a high PDE in the NIR band and a high FF.

Significant improvements have recently been achieved in the NIR PDE [5,6]. For example, the current state-of-the-art PDE of 20% at 870 nm has been obtained with an excess bias (*V_E_*) of 12 V [6]. Moreover, a fill factor of 21.6% has been reported [7]. This paper presents a new SPAD structure that achieves a similar PDE performance to that reported in [6], while also improving the sensitivity by optimizing the FF. Furthermore, the performance of this SPAD is described on the basis of various experimental results.

## 2. Design and Chip Implementation of NIR-Sensitivity-Enhanced SPADs

**Figure 1:**
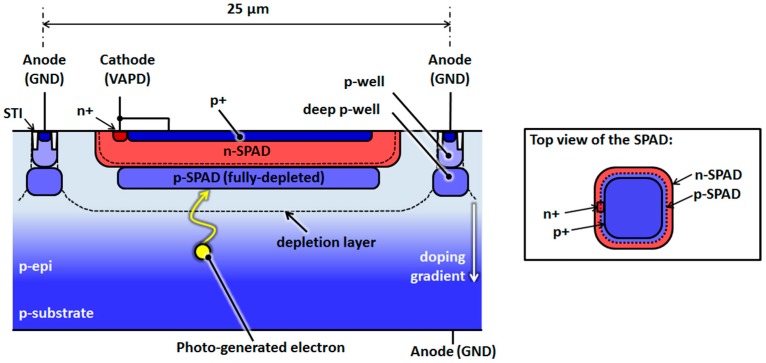
Simplified cross-sectional structure of the proposed SPAD. The top view of the SPAD is shown in the inset to illustrate how the SPAD forms an electrical contact with the n+ layer. Two custom layers, n-SPAD and p-SPAD, are employed for this SPAD, and the p-SPAD is fully depleted when the SPAD is biased at or above its breakdown voltage. Moreover, the p-epitaxial layer has a gradient doping profile. The thick depletion layer and doping profile efficiently collect electrons generated by the NIR light, which results in a high NIR PDE.

Figure 1 shows the cross-sectional structure of the newly designed SPAD, which achieves high sensitivity in the NIR band without compromising the FF. The SPAD lies in the p-epitaxial layer of a CMOS wafer and comprises two SPAD-specific custom layers, namely n-SPAD and p-SPAD. Isolation between adjacent SPADs (*i.e.*, a guard ring) is achieved by the superposition of existing p-well and deep p-well layers, which are available in most modern CMOS processes.

Since the absorption coefficient for NIR light is low compared to that for visible light, NIR light can penetrate deeper before it is completely absorbed in the silicon substrate. By increasing the vertical thickness of a depletion layer, the detection probability of incident NIR light is enhanced. The depletion layer thickness can be increased by increasing the SPAD bias voltage. However, a high bias voltage potentially leads to edge breakdown, which would interfere with the photon counting operation. To avoid edge breakdown and achieve high PDE in the NIR band, the distance between the SPAD and guard ring was increased, which reduces the FF. Conversely, a high-FF SPAD, achievable by thinning the depletion layer, has been proposed, which results in a lower PDE in the NIR band. Thus, previous SPAD designs have involved a tradeoff between the PDE and FF for sensing NIR lights, which represents an obstacle to higher sensitivity.

The proposed structure simultaneously achieves high PDE and FF. The doping concentrations of the two custom layers are optimized to obtain a high electric field for the avalanche multiplication of photo-generated electrons. Importantly, they are carefully designed so that the p-SPAD layer becomes fully depleted when the SPAD is biased at or above its breakdown voltage. Both a high electric field and a thick depletion layer are achieved under a low bias voltage, and thus, edge breakdown is avoided. As a result, the NIR PDE is enhanced without compromising the FF.

Furthermore, this structure also exploits a p-epitaxial layer that features a gradient doping profile; *i.e.*, a profile where the doping concentration increases with depth. Such a gradient doping profile further improves the PDE by promoting upward migration and efficient collection of photo-generated electrons toward the avalanche multiplication region. This technique has been used in image sensors to improve the quantum efficiency. It has also been reported in CMOS SPADs [5].

Additionally, the proposed SPAD also has a shallow p+ layer at the surface of the SPAD in order to collect surface-generated carriers, which reduces its intrinsic dark noise. Thus, the proposed structure prevents dark noise from triggering false avalanche events.

**Figure 2:**
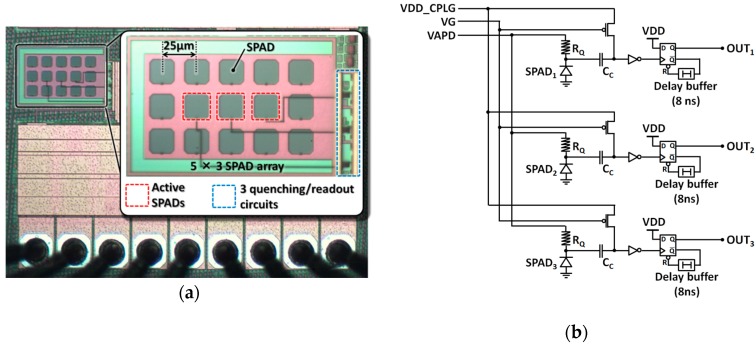
(**a**) Photomicrograph of a sample chip containing an array of 5 × 3 SPADs and three quenching/readout circuits. Only the innermost three SPADs are connected to the circuits. The remaining 12 SPADs are used as “dummy” devices; (**b**) a schematic of three quenching and readout circuits containing SPADs.

Figure 2a shows a photograph of a test chip that was fabricated to characterize the proposed SPAD using a 0.18-μm CMOS process. The test chip contains an array of 5 × 3 SPADs and three quenching/readout circuits. Only the innermost three SPADs are connected to these circuits, as shown in the figure, while the remaining 12 SPADs are used as dummies because the characteristics of the outermost devices in a device array are generally unreliable. The distance between adjacent SPADs is 25 μm. Figure 2b shows schematics of quenching and readout circuits containing the SPADs.

SPAD quenching and recharge is typically performed passively by high-resistivity polysilicon resistors of *R_Q_* = 300 kΩ. When a photon enters the SPAD, negative voltage pulses with an amplitude of approximately *V_E_* = *VAPD* − *V_BD_* are generated at the SPAD cathode, where *V_BD_* is the SPAD breakdown voltage, *VAPD* is the positive bias voltage, and *V_E_* is the SPAD excess bias. The generated pulse is then capacitively coupled via a 5 fF metal fringe capacitor (*C_C_*) to the input of a CMOS inverter that serves as a comparator. The inverter input is biased to *VDD_CPLG* by a thick-oxide PMOS transistor with a constant gate bias *VG*. The inverter is designed with thick-oxide transistors and is powered by the core 1.8 V supply voltage in this process. Positive pulses at the inverter output are transformed into rectangular pulses of approximately 8.5 ns by a D-flip-flop-based monostable circuit. This readout circuit imposes a minimum dead time of approximately 17 ns, regardless of the actual SPAD recharge time.

## 3. Experimental Results

**Figure 3:**
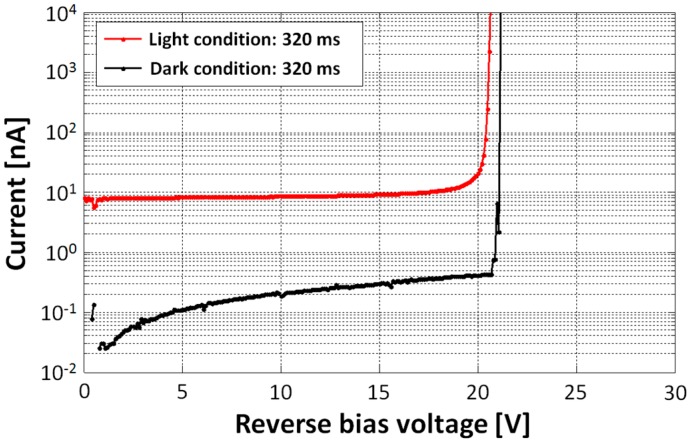
Static SPAD current characteristics as a function of the reverse bias voltage supplied to the SPAD cathode.

Figure 3 shows static SPAD current characteristics as a function of the reverse bias voltage supplied to the SPAD cathode. The current measurements are conducted under dark and light conditions with an integration time of 320 ms using a semiconductor parameter analyzer. From this result, the *V_BD_* of the proposed SPAD is approximately 20.5 V.

**Figure 4:**
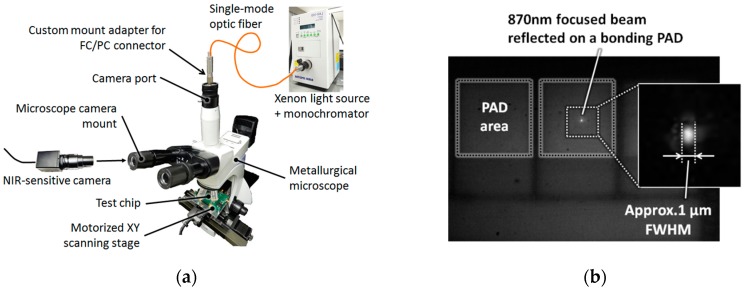
(**a**) Measurement system for high-resolution XY mapping of the photon response of a SPAD at any desired optical wavelength. An objective lens with an NA of 0.6 and ×40 is used in the microscope; (**b**) Image captured using an NIR-sensitive camera mounted on one of the microscope eyepieces.

Figure 4a shows the microscope-based measurement system used to accurately measure the FF of the proposed SPAD.

This system relies on the fact that all of the light coming out of a point source placed in the focal plane of the camera port is focused onto a point on the sample surface. Therefore, it provides an XY mapping of the photon counting rate of a SPAD at any desired optical wavelength with a high spatial resolution. In the microscope, an objective lens with an NA of 0.6 is used. Figure 4b shows the experimentally confirmed resolution of the proposed system and demonstrates that the spot size of an 870-nm focused beam is approximately 1 μm FWHM (full width at half maximum).

**Figure 5:**
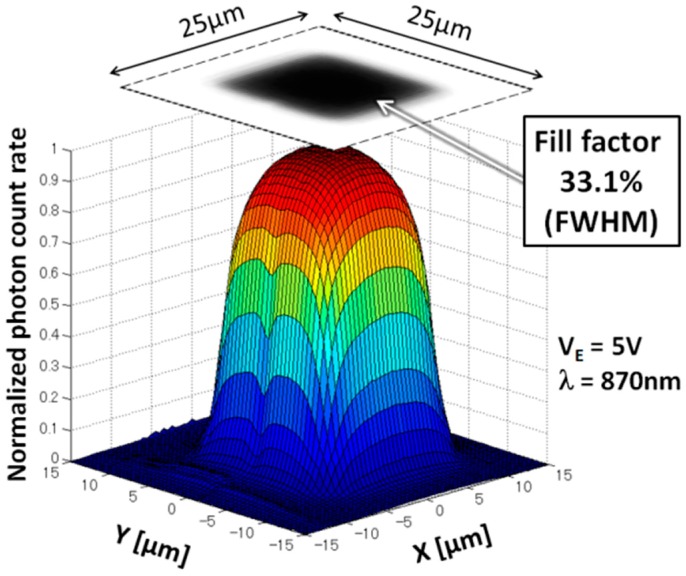
Normalized photon counting rate map of the proposed 25 × 25 μm^2^ SPAD. *V_E_* is 5 V and the incident cone of 870 nm light is approximately 74°.

Figure 5 shows a normalized map of the photon response of a SPAD with a resolution of 0.5 × 0.5 μm, using the system shown in Figure 4, measured with a *V_E_* of 5 V and 870-nm light. The measurement area is 30 × 30 μm, fully encompassing the 25 × 25 μm SPAD area. This result experimentally demonstrates that the proposed SPAD achieves a high FF of 33.1% FWHM.

**Figure 6:**
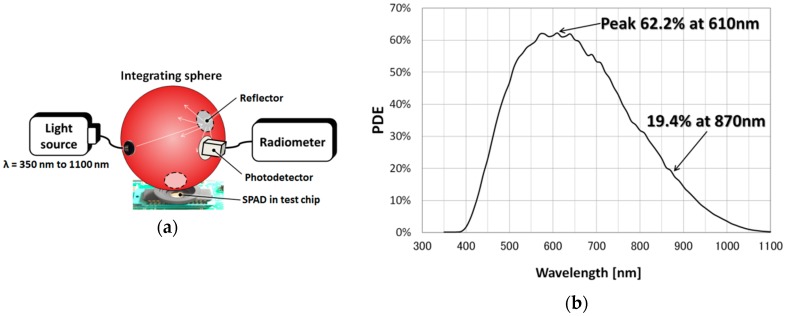
Measurement system (**a**) used to determine the PDE of the proposed SPAD. This system consists mainly of a light source, an integrating sphere, and a photodetector connected to a radiometer; (**b**) PDE as a function of light wavelength at an excess bias of 5 V.

Figure 6a shows a light source, an integrating sphere, and a reference photodiode based measurement system to measure the PDE of the proposed SPAD. A light source radiates light with wavelengths ranging from 350 to 1100 nm into the integrating sphere.

Figure 6b shows the measured PDE of the proposed SPAD as a function of light wavelength with a *V_E_* of 5 V. The proposed SPAD has a peak PDE of 62.2% at 610 nm and achieves a high PDE of 19.4% at 870 nm, which is the laser light wavelength of our LIDAR system. To the best of our knowledge, this is the highest PDE yet achieved for a CMOS SPAD under similar *V_E_* bias conditions.

**Figure 7:**
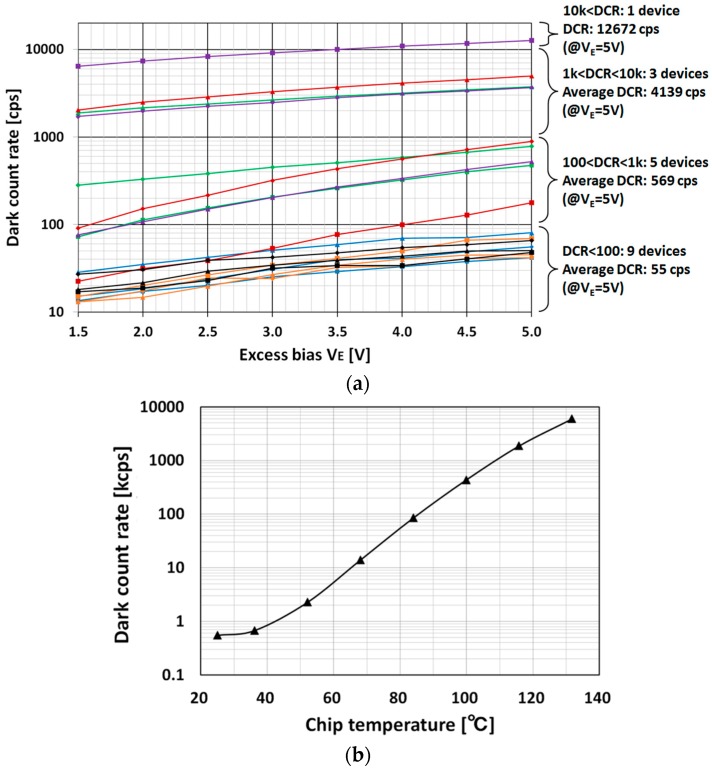
(**a**) Measurement results of DCR as a function of excess bias *V_E_*, using 18 SPAD samples in 6 test chips at room temperature; (**b**) Measurement results of DCR as a function of chip temperature, using one SPAD sample.

Figure 7 shows dark count rate (DCR) measurement results. The dark counts are caused by false output pulses, which are mainly induced by intrinsic dark noise in the SPAD, despite the absence of light incidence. A high DCR worsens the ranging performance, since false pulses obscure true pulses generated by the desired laser light. Figure 7a shows DCRs as a function of *V_E_* for 18 SPAD samples in six test chips at room temperature. As seen in this figure, the DCR values vary greatly among samples. Such large variances in DCR are common, as evidenced by [5,6]. At a *V_E_* of 5 V, DCRs of half of SPAD samples are less than 100 counts per second (cps), and the maximum DCR is 12.672 kcps, *i.e.*, false pulses are generated in cycles of 78.914 μs. However, these DCR values have little impact on the ranging performance. For example, since a 150 m ranging operation requires only 1 μs measurement time, dark counts (*i.e.*, false pulses) only affect one operation in approximately every 78 operations, even if the worst SPAD sample is used. Figure 7b shows the DCR as a function of chip temperature over the range of 25–132 °C. As the figure shows, the DCR grows exponentially with chip temperature, as dark noise is directly correlated with temperature.

The room-temperature DCR is less than 1 kcps. When the chip temperature increases, however, the DCR reaches 10 kcps at 65 °C, 100 kcps at 85 °C, 1 Mcps at 110 °C, and 6 Mcps at 132 °C. As noted previously, a 150 m ranging operation is completed in 1 μs, so the false pulse affects every ranging operation above 110 °C. However, our final chip will implement spatiotemporal correlation technology based on a macro-pixel structure [3], which will effectively suppress the impact of DCR at high temperatures. The present measurement results offer important insight for the application of the proposed SPAD to actual automotive systems that must operate at high temperatures.

**Figure 8:**
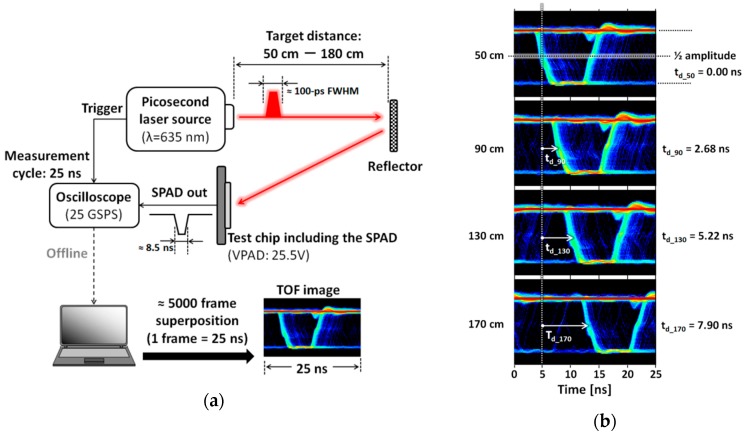
(**a**) Measurement system for the TOF and target distances; (**b**) TOF images superimposing 5000-frame SPAD out signals at 50, 90, 130, and 170 cm. From the TOF images, each delay time (*t_d_*) over various distances is measured, based on the result at 50 cm.

Figure 8a shows the elementary system used for measurements of the TOF and target distances using the proposed SPAD. Mainly, this experiment ensures that the proposed SPAD can accurately respond to narrow and high-speed laser pulses. This is a first step in the development of a LIDAR system. The present elementary system consists of a picosecond laser source (λ = 635 nm, FWHM ≈ 100 ps), a test chip containing SPADs to which a VAPD of 25.5 V is supplied, an oscilloscope (25 GSPS) to record trigger signals from the laser source and output signals of the SPAD with a period of 25 ns, a reflector as a measurement target, and an offline PC. When the pulsed laser light reflected by the reflector enters the SPAD in the test chip, a signal formed to an 8.5 ns PW is output from the chip. Finally, the PC superimposes approximately 5000 frames of the output signal data recorded by the oscilloscope and produces a TOF image similar to an eye diagram. In this measurement, the target distance ranges from 50 to 180 cm. Figure 8b shows representative TOF images at 50, 90, 130, and 170 cm. As seen in the figure, the pulse delay time (*t_d_*) based on the TOF at 50 cm increases linearly with increasing target distance.

**Figure 9:**
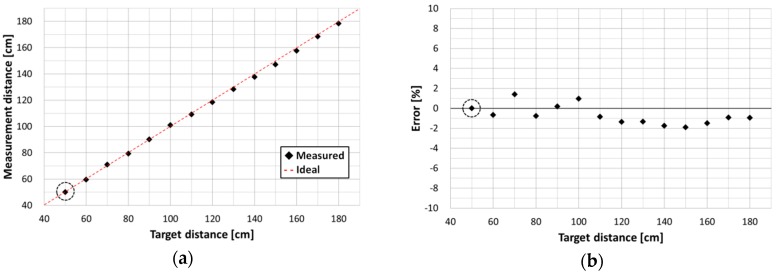
Measurement results of target distances at room temperature. (**a**) Measured distances to the target reflector as a function of actual target distance, based on the result obtained at 50 cm; (**b**) Measurement errors as a function of target distance, based on the result obtained at 50 cm.

Figure 9 shows measured distances to the target reflector at room temperature, based on the delay times (*t_d_*__50_–*t_d_*__180_) measured by the system in Figure 8. Figure 9a shows distances calculated from each *t_d_* as a function of target distance (the actual distance to the target reflector). As shown in the figure, the measured distances are comparable to the actual target distances. Figure 9b shows the error as a function of target distance. As seen in Figure 9b, the errors are all less than ±2%. These results demonstrate that the proposed SPAD can respond to narrow and high-speed laser pulses and can thus be used for ranging systems.

## 4. Conclusions

This paper presents a new SPAD structure to achieve a higher-performance, e.g., longer-range, higher-precision, and higher-measurement-rate, LIDAR system for future ADASs. In the proposed SPAD, two custom layers, p-SPAD and n-SPAD, are employed using the existing 0.18-μm CMOS technology. The doping concentrations of the customized layers are optimized to achieve a high electric field, while the p-type layer becomes fully depleted when the device is biased to operate in Geiger mode. This full-depletion layer enables the efficient collection of electrons generated by NIR light, without compromising the FF. Moreover, the p-epitaxial layer, which has a gradient doping profile, further enhances the NIR PDE.

In this study, various experiments are conducted to characterize the proposed SPAD. In SPAD PDE measurements, PDEs of 62.2% at 610 nm and 19.4% at 870 nm are attained. To the best of our knowledge, this is the highest PDE achieved for a CMOS SPAD under similar excess bias conditions, *i.e.*, 5 V. Moreover, the actual FF is measured by the special measurement system, and a high FF of 33.1% is obtained at 870 nm. Since SPAD sensitivity is characterized by the product of the PDE and FF, the proposed structure imparts the SPAD with higher NIR sensitivity, eliminating the PDE-FF tradeoff. The DCR levels of the proposed SPAD have only a small effect on the ranging performance, even when the worst SPAD among the test samples is used. The DCR measurement results at high temperature reveal new insight for actual vehicle installations. The ranging experiment demonstrates that target distances are successfully measured within an error of ±2%. We believe that the proposed SPAD will pave the way for higher-performance LIDAR and ADAS systems.
